# 4-Bromo-*N*-phenyl­aniline

**DOI:** 10.1107/S160053681101052X

**Published:** 2011-03-26

**Authors:** Emily J. Hefter, Joseph M. Tanski

**Affiliations:** a Department of Chemistry, Vassar College, Poughkeepsie, NY 12604, USA

## Abstract

In the title compound, C_12_H_10_BrN, the dihedral angle between the benzene rings is 52.5 (1)°, whereas the pitch angles, or the angles between the mean plane of each aryl group ‘propeller blade’ and the plane defined by the aryl bridging C—N—C angle, are 19.6 (2) and 36.2 (3)°. While the N—H group is not involved in hydrogen-bonding inter­actions, the structure exhibits a network of inter­molecular C—H⋯π and N—H⋯π inter­actions.

## Related literature

The title compound is an amine analogue of brominated diphenyl ether flame retardant materials commonly used in household items. For information on environmental and health concerns related to brominated flame retardants, see: de Wit (2002 ▶); Lunder *et al.* (2010 ▶). For the synthesis, see: He *et al.* (2008 ▶); Sus (1947 ▶). For a related structure and information on C—H⋯π and N—H⋯π inter­actions, see: Krzymiński *et al.* (2009 ▶). For a description of the pitch angle in similar diphenyl structures, see: Duong & Tanski (2011 ▶); Lim & Tanski (2007 ▶).
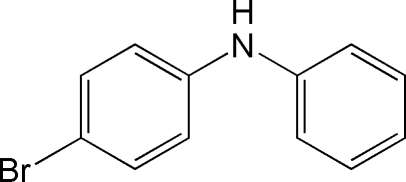


## Experimental

### 

#### Crystal data

C_12_H_10_BrN
*M* *_r_* = 248.12
Orthorhombic, 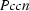

*a* = 15.6741 (6) Å
*b* = 17.7531 (7) Å
*c* = 7.3608 (3) Å
*V* = 2048.24 (14) Å^3^
*Z* = 8
Mo *K*α radiation
μ = 3.97 mm^−1^
*T* = 125 K
0.31 × 0.21 × 0.04 mm

#### Data collection

Bruker APEXII CCD diffractometer
Absorption correction: multi-scan (*SADABS*; Bruker 2007 ▶) *T*_min_ = 0.373, *T*_max_ = 0.857
31081 measured reflections
3137 independent reflections
2552 reflections with *I* > 2σ(*I*)
*R*_int_ = 0.038

#### Refinement

*R*[*F* ^2^ > 2σ(*F* ^2^)] = 0.043
*wR*(*F* ^2^) = 0.118
*S* = 1.07
3137 reflections
130 parameters
1 restraint
H atoms treated by a mixture of independent and constrained refinement
Δρ_max_ = 1.72 e Å^−3^
Δρ_min_ = −0.77 e Å^−3^

### 

Data collection: *APEX2* (Bruker, 2007 ▶); cell refinement: *SAINT* (Bruker, 2007 ▶); data reduction: *SAINT*; program(s) used to solve structure: *SHELXS97* (Sheldrick, 2008 ▶); program(s) used to refine structure: *SHELXL97* (Sheldrick, 2008 ▶); molecular graphics: *SHELXTL* (Sheldrick, 2008 ▶); software used to prepare material for publication: *SHELXTL* and *Mercury* (Macrae *et al.*, 2006 ▶).

## Supplementary Material

Crystal structure: contains datablocks I, global. DOI: 10.1107/S160053681101052X/om2415sup1.cif

Structure factors: contains datablocks I. DOI: 10.1107/S160053681101052X/om2415Isup2.hkl

Additional supplementary materials:  crystallographic information; 3D view; checkCIF report

## Figures and Tables

**Table 1:** C—H⋯π and N—H⋯π interactions (Å, °) *Cg*1 and *Cg*2 are the centroids of the C1–C6 and C7–C12 rings, respectively.

*D*—H⋯*A*	*D*—H	H⋯*A*	*D*⋯*A*	*D*—H⋯*A*
C6—H6⋯*Cg*2^i^	0.95	2.69	3.404 (3)	132
N1—H1⋯*Cg*1^ii^	0.85 (2)	2.65	3.501 (2)	175
C9—H9⋯*Cg*1^iii^	0.95	2.96	3.651 (3)	131

Symmetry codes: (i) 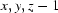; (ii) 
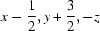
; (iii) 
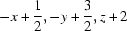
.

**Fig. 1.**
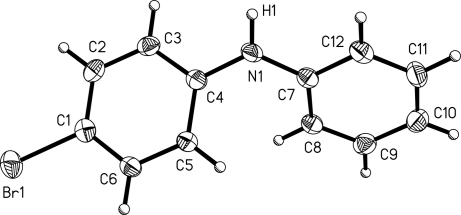
A view of the title compound, with displacement ellipsoids shown at the 50% probability level.

**Fig. 2.**
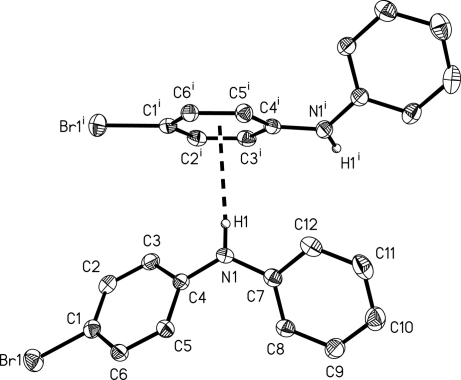
A view of the N—H···π intermolecular interaction with displacement ellipsoids shown at the 50% probability level [Symmetry codes: (i) x - 1/2, y + 3/2, -z].

## supplementary crystallographic information

### Comment

Diphenylamines have uses in chemical synthesis and materials chemistry, and they have been investigated for their biological activity (Krzymiński *et al.*, 2009). The title compound, a brominated diphenyl amine, was first synthesized by the photolysis of 4-diazodiphenylamine in the presence of HBr (Sus, 1947). More recently, halogenated diphenylamines have been prepared by copper catalyzed coupling reactions (He *et al.*, 2008). The title compound is an amine analogue of a class of brominated diphenyl ether materials (de Wit, 2002). Polybrominated diphenyl ethers are commonly used as flame retardants in consumer products and electronics and have been found in humans (Lunder *et al.*, 2010). The title compound is a monobrominated diphenyl amine derivative with a "propeller blade" disposition of the aryl rings about the bridging nitrogen atom. The aryl-bridging C4-N1-C7 angle is 126.4 (2)°, similar to the C-N-C bond angle of 126.1 (2)° found in the isomorphous structure of 4-methoxy-*N*-phenylaniline (Krzymiński *et al.*, 2009). The dihedral angle is found to be 52.5 (1)°, whereas the pitch angles are 19.6 (2)° and 36.2 (3)°. The pitch angles are the angles between the mean plane of each aryl group "propeller blade" and the plane defined by the three atoms C4-N1-C7 (Lim & Tanski, 2007; Duong & Tanski, 2011). In 4-methoxy-*N*-phenylaniline, the dihedral angle is somewhat larger, 59.9 (2)° (Krzymiński *et al.*, 2009), whereas the pitch angles, 7.2° and 53.8°, are very different, exemplifying how analogous structures with similar dihedral angles may have dramatically different dispostions of the aryl groups about the bridging atom.

The structure reveals that there is no intermolecular hydrogen bonding, however, a network of intermolecular C—H···π and N—H···π bonds exists (Table 1), as in the isomorphous structure of 4-methoxy-*N*-phenylaniline (Krzymiński *et al.*, 2009). These interactions are shorter in the title compound. The N—H···π centroid distance of 2.65 Å (Fig. 2) is shorter than the 2.88 Å distance observed in 4-methoxy-*N*-phenylaniline, and the N—H···π centroid angle of 175° is closer to linear than the 142° angle observed in 4-methoxy-*N*-phenylaniline, resulting in an interaction where the amine proton is directed at the center of the aromatic ring (Fig. 2), as opposed to at the edge of the ring as found the structure of 4-methoxy-*N*-phenylaniline.

### Experimental

Crystalline 4-bromo-*N*-phenylaniline was purchased from Aldrich Chemical Company, USA.

### Refinement

All non-hydrogen atoms were refined anisotropically. Hydrogen atoms on carbon were included in calculated positions and were refined using a riding model at C–H = 0.95Å and *U*_iso_(H) = 1.2 × *U*_eq_(C) of the aryl C-atoms. The hydrogen atom on nitrogen was refined semifreely with the help of a distance restraint, d(N–H) = 0.85 (2) Å and *U*_iso_(H) = 1.2 × *U*_eq_(N). There are three difference peaks > 1 e/Å^3^. The first and third highest difference peaks of 1.72 and 1.10 e/Å^3^ are < 0.8 Å from Br1, and the second highest difference peak of 1.64 e/Å^3^ is very close to the calculated H10 position. The extinction parameter (EXTI) refined to zero and was removed from the refinement.

### Crystal data

**Table:**

C_12_H_10_BrN	*F*(000) = 992
*M**_r_* = 248.12	*D*_x_ = 1.609 Mg m^−^^3^
Orthorhombic, *P**c**c**n*	Mo *K*α radiation, λ = 0.71073 Å
Hall symbol: -P 2ab 2ac	Cell parameters from 9926 reflections
*a* = 15.6741 (6) Å	θ = 2.3–30.2°
*b* = 17.7531 (7) Å	µ = 3.97 mm^−^^1^
*c* = 7.3608 (3) Å	*T* = 125 K
*V* = 2048.24 (14) Å^3^	Plate, colourless
*Z* = 8	0.31 × 0.21 × 0.04 mm

### Data collection

**Table:**

Bruker APEXII CCD diffractometer	3137 independent reflections
Radiation source: fine-focus sealed tube	2552 reflections with *I* > 2σ(*I*)
graphite	*R*_int_ = 0.038
φ and ω scans	θ_max_ = 30.5°, θ_min_ = 1.7°
Absorption correction: multi-scan (*SADABS*; Bruker 2007)	*h* = −22→22
*T*_min_ = 0.373, *T*_max_ = 0.857	*k* = −25→25
31081 measured reflections	*l* = −10→10

### Refinement

**Table:**

Refinement on *F*^2^	Primary atom site location: structure-invariant direct methods
Least-squares matrix: full	Secondary atom site location: difference Fourier map
*R*[*F*^2^ > 2σ(*F*^2^)] = 0.043	Hydrogen site location: inferred from neighbouring sites
*wR*(*F*^2^) = 0.118	H atoms treated by a mixture of independent and constrained refinement
*S* = 1.07	*w* = 1/[σ^2^(*F*_o_^2^) + (0.0513*P*)^2^ + 4.1415*P*] where *P* = (*F*_o_^2^ + 2*F*_c_^2^)/3
3137 reflections	(Δ/σ)_max_ = 0.001
130 parameters	Δρ_max_ = 1.72 e Å^−^^3^
1 restraint	Δρ_min_ = −0.77 e Å^−^^3^

### Special details

**Table:**

Experimental. A suitable crystal was mounted in a nylon loop with Paratone-N cryoprotectant oil.
Geometry. All e.s.d.'s (except the e.s.d. in the dihedral angle between two l.s. planes and the e.s.d. for hydrogen-pi acceptor interactions) are estimated using the full covariance matrix. The cell e.s.d.'s are taken into account individually in the estimation of e.s.d.'s in distances, angles and torsion angles; correlations between e.s.d.'s in cell parameters are only used when they are defined by crystal symmetry. An approximate (isotropic) treatment of cell e.s.d.'s is used for estimating e.s.d.'s involving l.s. planes. Esds for the hydrogen-pi acceptor interactions are taken as the e.s.d.'s on the hydrogen donor to mean plane of the pi-acceptor distances.
Refinement. Refinement of *F*^2^ against ALL reflections. The weighted *R*-factor *wR* and goodness of fit *S* are based on *F*^2^, conventional *R*-factors *R* are based on *F*, with *F* set to zero for negative *F*^2^. The threshold expression of *F*^2^ > σ(*F*^2^) is used only for calculating *R*-factors(gt) *etc*. and is not relevant to the choice of reflections for refinement. *R*-factors based on *F*^2^ are statistically about twice as large as those based on *F*, and *R*- factors based on ALL data will be even larger.

### Fractional atomic coordinates and isotropic or equivalent isotropic displacement parameters (Å^2^)

**Table:**

	*x*	*y*	*z*	*U*_iso_*/*U*_eq_	
Br1	0.14415 (2)	0.595132 (18)	0.26943 (4)	0.03453 (12)	
N1	−0.02798 (16)	0.68765 (12)	0.9753 (3)	0.0256 (5)	
H1	−0.009 (2)	0.7273 (14)	1.025 (4)	0.031*	
C1	0.09072 (18)	0.62304 (14)	0.4908 (4)	0.0237 (5)	
C2	0.13506 (17)	0.66570 (15)	0.6173 (4)	0.0250 (5)	
H2	0.1920	0.6811	0.5936	0.030*	
C3	0.09542 (18)	0.68562 (15)	0.7787 (4)	0.0248 (5)	
H3	0.1258	0.7148	0.8654	0.030*	
C4	0.01153 (17)	0.66358 (14)	0.8166 (3)	0.0220 (5)	
C5	−0.03227 (17)	0.62058 (14)	0.6857 (3)	0.0225 (5)	
H5	−0.0893	0.6052	0.7082	0.027*	
C6	0.00745 (18)	0.60043 (14)	0.5232 (3)	0.0228 (5)	
H6	−0.0224	0.5714	0.4353	0.027*	
C7	−0.08697 (16)	0.64678 (14)	1.0792 (3)	0.0220 (5)	
C8	−0.09352 (17)	0.56827 (14)	1.0688 (4)	0.0233 (5)	
H8	−0.0593	0.5412	0.9846	0.028*	
C9	−0.14950 (19)	0.52990 (17)	1.1805 (4)	0.0294 (6)	
H9	−0.1534	0.4766	1.1721	0.035*	
C10	−0.2008 (2)	0.5685 (2)	1.3063 (4)	0.0343 (6)	
H10	−0.2389	0.5419	1.3835	0.041*	
C11	−0.19422 (19)	0.6463 (2)	1.3148 (4)	0.0338 (6)	
H11	−0.2284	0.6733	1.3993	0.041*	
C12	−0.13888 (18)	0.68566 (16)	1.2028 (4)	0.0271 (5)	
H12	−0.1361	0.7390	1.2098	0.033*	

### Atomic displacement parameters (Å^2^)

**Table:**

	*U*^11^	*U*^22^	*U*^33^	*U*^12^	*U*^13^	*U*^23^
Br1	0.03463 (18)	0.03743 (18)	0.03153 (17)	0.00533 (12)	0.00963 (12)	−0.00285 (11)
N1	0.0360 (12)	0.0194 (9)	0.0213 (10)	−0.0046 (9)	0.0032 (9)	−0.0044 (8)
C1	0.0275 (12)	0.0196 (11)	0.0241 (12)	0.0050 (9)	0.0014 (10)	0.0036 (9)
C2	0.0217 (12)	0.0233 (11)	0.0301 (13)	−0.0011 (9)	−0.0001 (10)	0.0059 (10)
C3	0.0286 (12)	0.0211 (11)	0.0246 (12)	−0.0041 (9)	−0.0039 (10)	0.0020 (9)
C4	0.0299 (13)	0.0175 (10)	0.0187 (11)	−0.0007 (9)	−0.0005 (9)	0.0014 (8)
C5	0.0238 (12)	0.0238 (11)	0.0200 (11)	−0.0010 (9)	−0.0011 (9)	−0.0012 (9)
C6	0.0283 (12)	0.0203 (11)	0.0198 (11)	0.0000 (9)	−0.0017 (9)	−0.0003 (9)
C7	0.0233 (12)	0.0232 (11)	0.0194 (11)	0.0023 (9)	−0.0033 (9)	−0.0004 (9)
C8	0.0255 (12)	0.0219 (11)	0.0225 (11)	0.0024 (9)	−0.0021 (9)	−0.0016 (9)
C9	0.0315 (14)	0.0302 (13)	0.0266 (13)	−0.0056 (11)	−0.0024 (11)	0.0023 (10)
C10	0.0287 (14)	0.0461 (17)	0.0280 (14)	−0.0090 (13)	0.0008 (11)	−0.0012 (12)
C11	0.0255 (13)	0.0497 (18)	0.0263 (13)	0.0019 (12)	0.0033 (11)	−0.0095 (12)
C12	0.0275 (13)	0.0283 (13)	0.0257 (12)	0.0046 (10)	−0.0027 (10)	−0.0053 (10)

### Geometric parameters (Å, °)

**Table:**

Br1—C1	1.898 (3)	C6—H6	0.9500
N1—C4	1.389 (3)	C7—C8	1.400 (4)
N1—C7	1.402 (3)	C7—C12	1.402 (4)
N1—H1	0.849 (18)	C8—C9	1.382 (4)
C1—C6	1.386 (4)	C8—H8	0.9500
C1—C2	1.387 (4)	C9—C10	1.404 (4)
C2—C3	1.387 (4)	C9—H9	0.9500
C2—H2	0.9500	C10—C11	1.388 (5)
C3—C4	1.400 (4)	C10—H10	0.9500
C3—H3	0.9500	C11—C12	1.385 (4)
C4—C5	1.408 (4)	C11—H11	0.9500
C5—C6	1.395 (4)	C12—H12	0.9500
C5—H5	0.9500		
			
C4—N1—C7	126.4 (2)	C5—C6—H6	120.2
C4—N1—H1	117 (2)	C8—C7—N1	122.3 (2)
C7—N1—H1	115 (2)	C8—C7—C12	118.9 (2)
C6—C1—C2	121.0 (2)	N1—C7—C12	118.8 (2)
C6—C1—Br1	119.2 (2)	C9—C8—C7	120.3 (3)
C2—C1—Br1	119.9 (2)	C9—C8—H8	119.8
C3—C2—C1	119.3 (2)	C7—C8—H8	119.8
C3—C2—H2	120.3	C8—C9—C10	121.0 (3)
C1—C2—H2	120.3	C8—C9—H9	119.5
C2—C3—C4	121.4 (2)	C10—C9—H9	119.5
C2—C3—H3	119.3	C11—C10—C9	118.2 (3)
C4—C3—H3	119.3	C11—C10—H10	120.9
N1—C4—C3	120.0 (2)	C9—C10—H10	120.9
N1—C4—C5	121.6 (2)	C12—C11—C10	121.4 (3)
C3—C4—C5	118.2 (2)	C12—C11—H11	119.3
C6—C5—C4	120.5 (2)	C10—C11—H11	119.3
C6—C5—H5	119.7	C11—C12—C7	120.1 (3)
C4—C5—H5	119.7	C11—C12—H12	120.0
C1—C6—C5	119.6 (2)	C7—C12—H12	120.0
C1—C6—H6	120.2		
			
C6—C1—C2—C3	0.3 (4)	C4—C5—C6—C1	0.0 (4)
Br1—C1—C2—C3	−179.87 (19)	C4—N1—C7—C8	20.8 (4)
C1—C2—C3—C4	0.0 (4)	C4—N1—C7—C12	−161.7 (3)
C7—N1—C4—C3	−145.7 (3)	N1—C7—C8—C9	176.6 (3)
C7—N1—C4—C5	38.0 (4)	C12—C7—C8—C9	−0.8 (4)
C2—C3—C4—N1	−176.7 (2)	C7—C8—C9—C10	0.0 (4)
C2—C3—C4—C5	−0.2 (4)	C8—C9—C10—C11	0.4 (4)
N1—C4—C5—C6	176.6 (2)	C9—C10—C11—C12	0.1 (4)
C3—C4—C5—C6	0.3 (4)	C10—C11—C12—C7	−1.0 (4)
C2—C1—C6—C5	−0.3 (4)	C8—C7—C12—C11	1.3 (4)
Br1—C1—C6—C5	179.89 (19)	N1—C7—C12—C11	−176.2 (3)

### Hydrogen-bond geometry (Å, °)

**Table:**

Cg1 and Cg2 are the centroids of the C1–C6 and C7–C12 rings, respectively.

**Table:**

*D*—H···*A*	*D*—H	H···*A*	*D*···*A*	*D*—H···*A*
C6—H6···Cg2^i^	0.95	2.69	3.404 (3)	132
N1—H1···Cg1^ii^	0.85 (2)	2.65	3.501 (2)	175
C9—H9···Cg1^iii^	0.95	2.96	3.651 (3)	131

Symmetry codes: (i) *x*, *y*, *z*−1; (ii) *x*−1/2, *y*+3/2, −*z*; (iii) −*x*+1/2, −*y*+3/2, *z*+2.
